# Immunodiagnosis of cattle fascioliasis using a 27 kDa *Fasciola gigantica* antigen

**DOI:** 10.14202/vetworld.2021.2097-2101

**Published:** 2021-08-16

**Authors:** Mohamed J. Saadh, Samer A. Tanash, Ammar M. Almaaytah, Issam J. Sa’adeh, Saed M. Aldalaen, Khawla D. Al-Hamaideh

**Affiliations:** 1 Department of Pharmacy, Faculty of Pharmacy, Middle East University, Amman, Jordan; 2 Department of Pharmaceutical Technology, Faculty of Pharmacy, Jordan University of Science and Technology, Irbid, Jordan; 3 Department of Radiology, King Abdulaziz Medical City, National Guard Hospital, Riyadh, Saudi Arabia; 4 Department of Pharmacology Faculty of Pharmacy, Mutah University, Amman, Jordan; 5 Department of Basic Medical Sciences, Faculty of Medicine, Al-Balqa Applied University, Amman, Jordan

**Keywords:** antigen, *Fasciola gigantica*, fascioliasis, immunodiagnostics

## Abstract

**Background and Aim:**

Diagnosis of fascioliasis depends on clinical symptoms and routine laboratory tests. Recently, antibodies and circulating antigens of Fasciola were used for detecting active infections. Therefore, this study aimed to identify Fasciola gigantica antigens in the sera of infected cattle using Western blotting and enzyme-linked immunosorbent assay (ELISA) for an accurate diagnosis of cattle infected with F. gigantica.

**Materials and Methods:**

Serum samples were obtained from 108, 23, and 19 cattle infected with Fasciola gigantica, Paramphistomum cervi, and Strongylids, respectively, including 57 non-infected cattle that were used as healthy cattle for the study. Western blotting and ELISA were then used to detect circulating Fasciola antigens at 27 kDa.

**Results:**

The target epitope was detected in an F. gigantica adult-worm antigen preparation, excretory/secretory products, and serum from cattle infected with F. gigantica. However, it was absent in sera from P. cervi, Strongylids, and healthy cattle. The purified 27 kDa F. gigantica (FPA-27) antigen was also detected in cattle serum using ELISA with high degrees of sensitivity and specificity (94% and 82%, respectively), and the area under the receiver operating characteristic curve was 0.89 with a highly significant correlation of p<0.0001.

**Conclusion:**

The FPA-27 is proposed to be a promising candidate for the serodiagnosis of fascioliasis in cattle.

## Introduction

Fascioliasis is a global zoonotic disease caused by liver trematodes of the genus *Fasciola*. The disease is recognized as an important infectious condition by the World Health Organization. Fascioliasis is also the most widespread parasitic disease of ruminants worldwide. Furthermore, it affects humans in 51 countries across several continents, including up to 17 million people, and more than 180 million people are at risk worldwide [1,2], thereby causing important economic losses to sheep and cattle in commercial herds [3,4]. Cattle infections usually occur by the ingestion of aquatic plants that contain the infective metacercariae [5].

Diagnosis of fascioliasis depends on clinical symptoms and routine laboratory tests. The most reliable diagnostic method is the presence of eggs in the stool of infected individuals. However, despite its reliability, an overwhelming consensus exists that this method is not completely reliable [6]. Disadvantages of this method include that eggs are not detected until a late stage of infection when liver damage has occurred. Furthermore, eggs are secreted from the bile ducts intermittently; therefore, stool samples from these infected cattle do not contain eggs [6,7]. On the basis of these limitations, early serological diagnosis is preferred by detecting anti-*Fasciola* antibodies to facilitate early therapeutic interventions because it appears 2 weeks after infection [8]. Lately, 26-28 kDa antigens in sera of infected cattle have been identified [9]. It was characterized as a protein containing 47.5% and 29.3% hydrophilic and hydrophobic amino acids, respectively. Immunostaining then demonstrated that the target epitope was located in the gut and tegument of adult *Fasciola gigantica* and within the bile ducts, portal tracts of the livers, and the mucosa, including the muscularis of the gallbladder of infected cattle. A simple and rapid dot enzyme-linked immunosorbent assay (ELISA) technique depending on the rabbit anti-serum was also developed. Furthermore, the 27 kDa form of the adult’s excretory/secretory (E/S) product gave a consistent reaction with fascioliasis from human sera [7].

Therefore, this study aimed to identify *F. gigantica* antigens in the sera of infected cattle using ELISA for an accurate diagnosis of cattle infected with *F. gigantica*.

## Materials and Methods

### Ethical approval

The study was performed in accordance with the guidelines of the National Council for Animal Experimentation Control, and the ethical committee approval was obtained from Ethical Committee of Middle East University, Jordan (Approval no. MEU5/3).

### Study period and location

This study was conducted from March 2018 to May 2019 on an animal farm in Dhail, Jordan.

### Sample collection

In this study, 207 cattle were included. Accordingly, all cattle were diagnosed using parasitological methods for demonstrating *F. gigantica*, *Paramphistomum cervi*, and *Strongylids* eggs in their stools. Briefly, 5 g of stool homogenate was incubated and suspended for 1 h in 20 mL of 0.9% (m/V) NaCl. Then, the stool suspension was filtered into a cone glass through a screen (aperture: 250 μm) coated with a triple-layered medical gauze. Subsequently, the rinse residue was conducted with 108 mL of 0.9% (m/V) NaCl. For the sedimentation of *F. gigantica*, *P. cervi*, and *Strongylids* eggs, the suspension was permitted to stand for 1 h. After that time, the supernatant was decanted into a 15 mL conical tube, and the sediment was transferred and weighed. Next, the eight sediment samples (30 μL per sample) were placed under coverslips (21×26 mm) on microscope slides and analyzed under a light microscope (Axiolab 5 Pol, USA) at 100×. *Fasciola* eggs present appeared small and had a yellow coloration. The 108 cattle had *F. gigantica* eggs in their feces, 23 cattle had *P. cervi*, and 19 cattle had *Strongylids*. The remaining 57 cattle were healthy with no parasitic infection.

### Parasite antigenic preparations

The crude E/S. *P. cervi* worm antigen was prepared following the same procedure as *F. gigantica* worm antigen preparations (FWAP) that described by Attallah *et al*.[10]

### Sodium dodecyl sulfate-polyacrylamide gel electrophoresis (SDS-PAGE) and gel electroelution

Different samples underwent SDS-PAGE with 50 μg/lane vertical slabs of 12% or 16% polyacrylamide each. Protein molecular weight standards (Sigma-Aldrich, Saint Louis, MO, USA) were subsequently run in parallel [9]. Next, Coomassie brilliant blue R 250 (Sigma-Aldrich) was added to the setup, after which the 27 kDa band was cut from the gel, and the antigens were electroeluted at 200 V for 3 h in a dialysis bag from the polyacrylamide gels (Sigma-Aldrich). After dialysis, the electroeluted antigens were purified and concentrated as assays following the previously described methods [9]. Finally, the purified 27 kDa *F. gigantica* (FPA-27) antigen content of the electroeluted antigen sample was determined and then stored at −20°C.

### Western blots

For this analysis, samples separated on SDS-PAGE were electrotransferred onto nitrocellulose (NC) membranes (0.45 μm pore size; Sigma-Aldrich) in a protein transfer unit. The NC membrane was then blocked and rinsed as described previously [10]. Next, the membrane was incubated with anti-FPA-27 immunoglobulin G (IgG) antibodies, diluted (1:100) in a blocking buffer with steady shaking. The blots were washed 3 times (30 min each) in Tris-buffered saline (TBS) followed by further incubation for 2 h with diluted 1:350 anti-rabbit IgG alkaline phosphatase conjugate in TBS (Sigma-Aldrich). After washing 3 more times with TBS (15 min each), the blots were soaked in the substrate. Then, the color reaction was observed within 10 min and stopped by dipping the blots in distilled water.

### Production of 27 kDa *anti-Fasciola* specific antibody

Anti-*Fasciola* IgG polyclonal antibodies were produced and used according to the procedure by Attallah *et al*. [9]. Briefly, a rabbit was immunized with crude FPA-27. Then, equal volumes of antigen (500 μg/mL) and complete Freund’s adjuvant (CFA) or incomplete Freund’s adjuvant (IFA) were homogenized together. Next, each rabbit was subcutaneously inoculated 3 times: Once with antigen in CFA (1 mL on day 0) and twice with antigen in IFA (1 mL each on days 15 and 28) before being sacrificed and exsanguinated on day 32. Subsequently, the sera were separated, labeled, and stored at −20°C until use.

### Detection of 27 kDa *Fasciola antigens* in the serum using ELISA

Polystyrene flat-bottom microtiter plates (Coster, Acton, MA, USA) were coated with 1:200 diluted cattle serum samples in carbonate-bicarbonate buffer, pH 9.6. After blocking, 50 μL (per well) of 1:200 dilution in phosphate-buffered saline (PBS) with 0.05% (v/v) Tween 20 (PBS-T20) of anti-FPA-27 IgG antibody was added. Then, the plates were incubated at 37°C for 2 h. After washing, 50 mL (per well) of anti-rabbit IgG alkaline phosphatase conjugates (whole molecule; Sigma-Aldrich), diluted at 1:600 with 0.2% (wt/v) BSA in PBS-T20, was added. Next, the plate was incubated at 37°C for 1 h. Furthermore, after washing, the substrate (p-nitrophenyl phosphate in 0.1 M glycine buffer; pH 10.4) was added, and the plates were incubated for 30 min at 37°C. ODs were then read at 490 nm using a microplate autoreader (Metertech Inc., Taiwan).

For determination, the 16 cutoff serum samples from healthy individuals, in addition to eight serum samples from fascioliasis showing eggs in stools, were used. Then, the cutoff level for ELISA was determined as the mean ELISA ODs of serum samples from 16 healthy cattle + three standard deviations. The cutoff was equal to 0.34.

### Statistical analysis

Data were presented as mean±SD for the three parallel measurements. Statistical significance was also assessed through t-test (using two-tailed distribution) using Statistical Package for the Social Sciences (SPSS) software version 20.0 (SPSS Inc., Chicago, IL, USA). p<0.05 was set as statistical significance.

## Results

### Identification of the FPA-27 among antigenic extracts from *F. gigantica*

**Figure-1:**
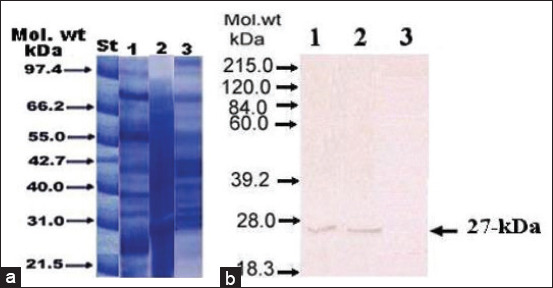
(a) Sodium dodecyl sulfate-polyacrylamide gel electrophoresis. (b) Western blotting for different antigenic sources of *Fasciola gigantica* and *Paramphistomum cervi* using immunoglobulin G rabbit anti-*Fasciola* antibody. Lane 1: *F. gigantica* adult worm antigen preparation; lane 2: Excretory/secretory antigen products from *F. gigantica*; lane 3: *P. cervi* antigenic extract.

FWAP and E/S results of *F. gigantica* and *P. cervi* antigens were subjected to SDS-PAGE (Figure-1a), after which *Fasciola* antigen was detected by Western blotting. It was found that the 27 kDa polypeptide band in both the FWAP and E/S reacted with anti-FPA-27 IgG antibody (Figure-1b). However, this anti-FPA-27 antibody did not recognize any antigen in *P. cervi* antigenic extract (Figure-1b).

### Identification of the 27 kDa target antigen in the cattle serum sample from cattle infected with *F. gigantica* using SDS-PAGE and Western blotting

**Figure-2:**
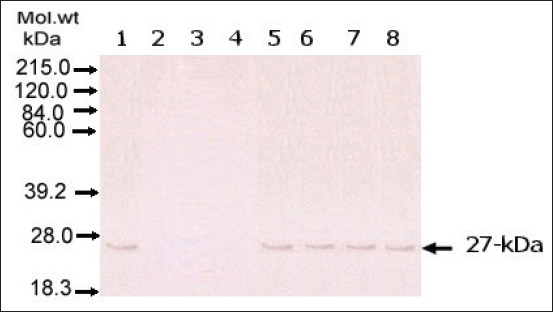
Western blot of serum samples from *Fasciola*-infected and non-infected cattle to detect circulating *Fasciola* antigen. Lane 1: *Fasciola* *gigantica* worm antigen preparations (*F. gigantica*); lane 2: Serum sample from non-infected cattle; lane 3: Serum sample from cattle infected with *Paramphistomum cervi*; lane 4: Serum sample from cattle infected with *Strongylids*; lane 5-8: Serum samples from cattle infected with *F. gigantica*.

An intense, sharp band was seen at 27 kDa in the sera from cattle infected with *F. gigantica* and FWAP, but no sharp band was seen in both healthy cattle and uninfected cattle with fascioliasis (Figure-2).

### Detection of the 27 kDa *F. gigantica* antigen in serum samples using ELISA

**Figure-3:**
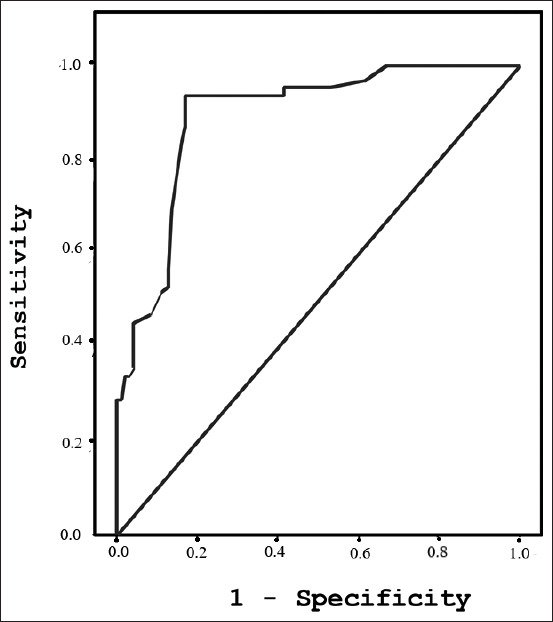
Area under the receiver operating characteristic curve for diagnostics of *Fasciola gigantica* against all samples from cattle (area under curve=0.89; p<0.0001).

**Figure-4:**
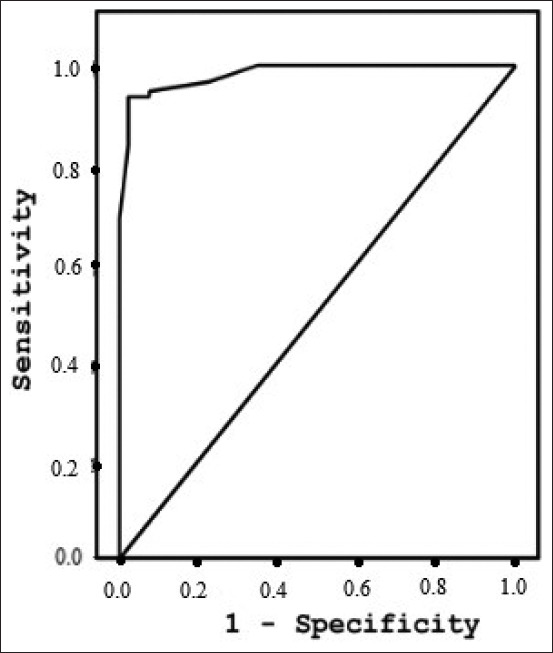
Area under the receiver operating characteristic curve for diagnostics of *Fasciola gigantica* versus healthy cattle (area under curve=0.98; p<0.0001).

Serum samples from 108 cattle with fascioliasis, 23 cattle with *P. cervi*, 19 cattle with *Strongylids*, and 57 non-infected, healthy cattle were tested to detect circulating *Fasciola* antigen. ELISA successfully detected circulating 27 kDa *F. gigantica* antigen in the serum samples. ELISA also detected fascioliasis with a sensitivity of 94% and a specificity of 82%. The positive and negative predictive values (PPV and NPV, respectively) were 77% and 95%, respectively, and the efficiency was 87%. Furthermore, the area under the receiver operating characteristic curve area under curve (AUC) was 0.89, with a highly significant correlation of p<0.0001 (Figure-3). Therefore, when cattle infected with *P. cervi* and *Strongylids* were excluded, the AUC for 27 kDa *F. gigantica* antigen in the serum from fascioliasis cattle versus healthy cattle was increased to 0.98 (Figure-4), with 94% sensitivity, 95% specificity, and a highly significant correlation (p<0.0001).

## Discussion

*Fasciola* antigens are excreted from the gut and tegument into the bloodstream. Several circulating antigens in the serum for *F. gigantica* have been detected in feces and were used in immunodiagnosis of fascioliasis in cattle. Therefore, *F. gigantica* antigens at 27 kDa have been identified in FWAP, E/S products, and human serum, and these antigens are of crucial importance for diagnosis and further development of a molecular-based vaccine [11,12].

Comparison with other methods that can identify samples from cattle with pre-patent or early infections distinguishes antigen detection, which is undetectable by the usual parasitological tests. In addition, these assays give a more accurate indication of the current state of infections [13]. In a previous study, Attallah *et al*. [10] identified a band at 27 kDa during E/S antigenic examinations in the adult worm of *F. gigantica* and the sera of humans infected with *F. gigantica*.

Therefore, in this study, the IgG antibody in rabbits was produced using FPA-27 by isolating their 27 kDa antigens and purifying serum samples from cattle infected with *F. gigantica*. Results showed that the antigen of 27 kDa was identified in adult FWAP and E/S products of *F. gigantica* using IgG antibodies but did not recognize epitopes in *P. cervi* and *Strongylids* antigenic extracts. *F. gigantica* antigen described as 27 kDa *F. gigantica* was also similar in molecular mass to the antigen described in a similar study by Attallah *et al*. [10]. The biochemical characterization of 26–28 kDa of *F. gigantica* antigens also confirmed its protein moiety [9].

Furthermore, our study describes the detection of FPA-27 in cattle serum using ELISA as a diagnostic tool. The results indicated that the overall sensitivity of this assay for detecting 27 kDa *F. gigantica* antigens was 94% among proven *F. gigantica*-infected cattle, with a specificity of 82%. Another study showed similar sensitivity (94.5%) and lower specificity (84.6%) using ELISA sandwiches based on a monospecific rabbit IgG antibody against 14.5 kDa protein antigens obtained from *F. gigantica* adult worms [14,15].

The cross-reactivity with other parasites has also been reported, decreasing the specificity of the techniques, especially in areas of disease endemicity [16,17]. Regarding the specificity of the developed FPA-27 ELISA, there were no major problems related to cross-reactivity with *P. cervi* and *Strongylids*. This finding was not strange because the specificity of the FPA-27 ELISA depended mainly on the recognition of a single *Fasciola* antigen (27 kDa) by a monospecific IgG antibody. Serological tests for antibodies have limited diagnostic value as well because antibody titers persist after the infected cattle have been cured. In addition, the infected cattle with recent infections had a negative test; a phenomenon demonstrated in experimental animals during the first 1-4 weeks of infection [18-20].

## Conclusion

The FPA-27 was identified in sera of *F. gigantica*-infected cattle. Therefore, we propose that the FPA-27 for the early diagnosis of *F. gigantica* in cattle is simple, rapid, sensitive, and specific.

## Authors’ Contributions

MJS, SAT, SMA, and AMA: Designed the study and drafted the manuscript. MJS, IJS, KDA, AMA, and SMA: Performed all the experimental procedures. MJS, KDA, and IJS: Conducted data analysis and interpretation. All authors read and approved the final manuscript.
